# Supplementary material for: Transferrin receptor 1 is a cellular receptor for human heme-albumin
Source: Commun Biol. 2020 Oct 27;3:621. doi: 10.1038/s42003-020-01294-5 (PMC7591885; doi:10.1038/s42003-020-01294-5)
Supplement: Supplementary file 1 — Supplementary Information [file 42003_2020_1294_MOESM1_ESM.pdf]

Supplementary Figure 1

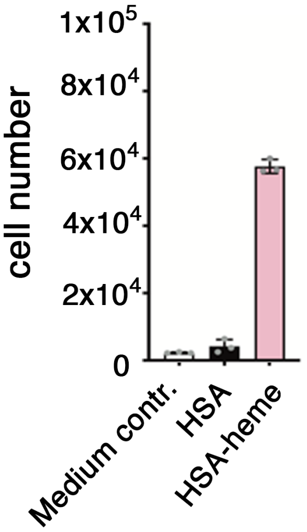

Recombinant human serum albumin (rHSA) bound to heme promotes proliferation of Jurkat T cells. Cells were cultivated with rice rHSA or rice rHSA loaded with heme (both, 200 µg/ml) in comparison to serum- and protein-free medium (Medium contr.). Error bars represent standard deviation (SD) of one experiment out of two analyzed in triplicates.

Supplementary Figure 2

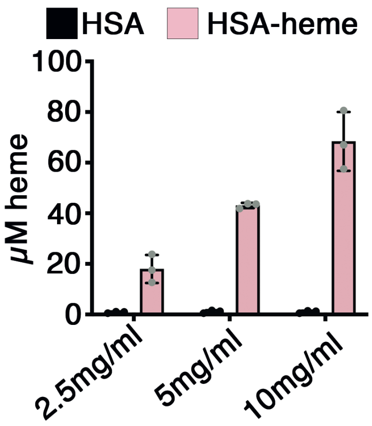

Quantification of heme concentration in HSA. The bars illustrate the amount of heme bound to HSA and HSA-heme. Proteins were titrated and the protein bound heme concentration was measured by a Heme Assay Kit. Results are displayed standard error of the mean (SEM) of three independent experiments.

Supplementary Figure 3

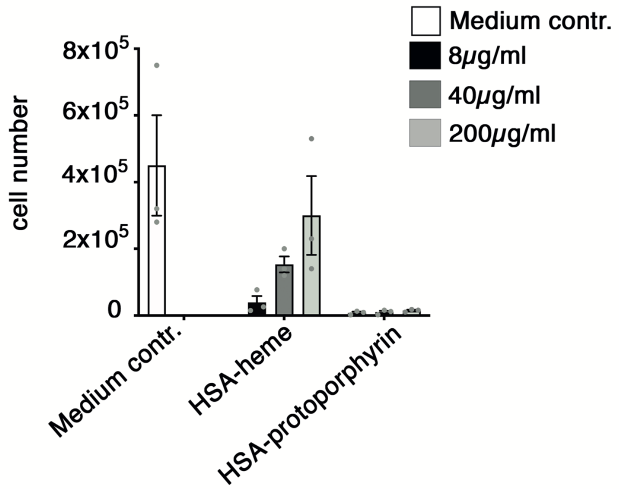

Effect of protoporphyrin bound to HSA on cell proliferation. Jurkat T cells were cultured in protein- and serum-free medium supplemented with HSA-heme, HSA-protoporphyrin at different concentrations or 10% FCS (Medium contr.) Bars indicate mean ± SEM of three independent experiments.

Supplementary Figure 4

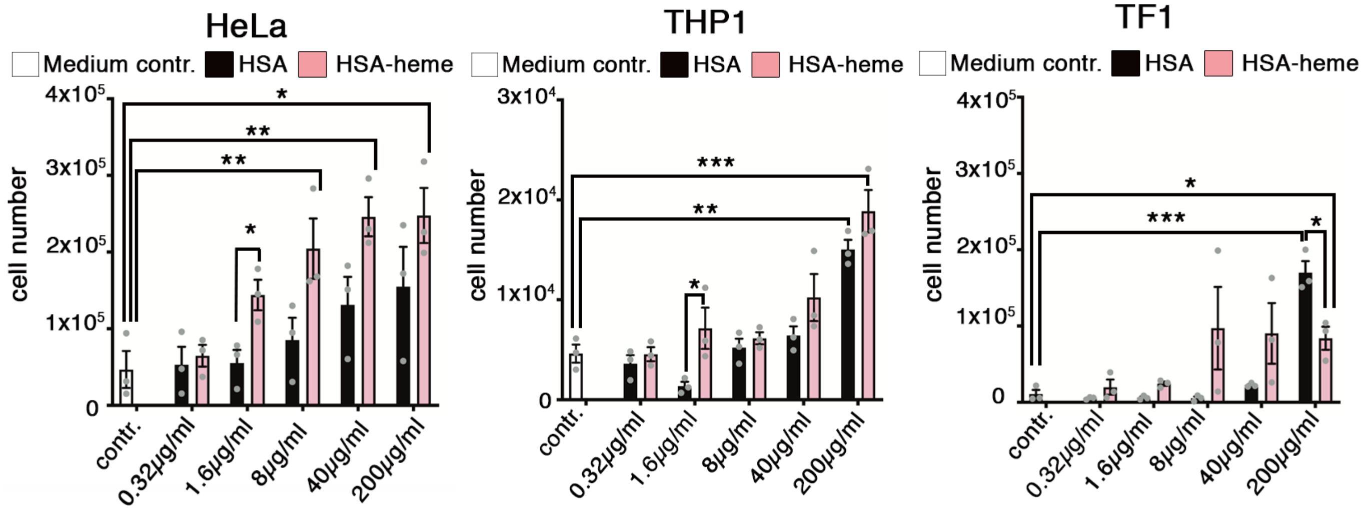

Proliferation effect of HSA-heme on different cell lines. Comparison of cell growth in presence of HSA or HSA-heme at different concentrations in comparison to serum – and protein-free medium (contr.) Data are displayed as mean ± SEM of three independent experiments. Statistics by one-way ANOVA, followed by Tukey's multiple comparison test. $^{*}p < 0.05$, $^{**}p > 0.01$, $^{***}p < 0.001$.

Supplementary Figure 5

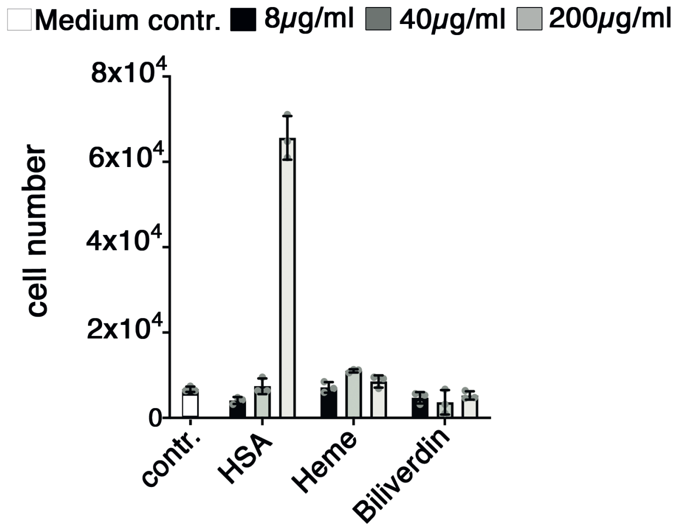

Effect of free heme and biliverdin on cell proliferation. Jurkat T cells were cultured in serum- and protein-free medium (contr.) supplemented with HSA, heme or biliverdin at different concentrations. Bars indicate mean ± SD of triplicates of one experiment.

Supplementary Figure 6

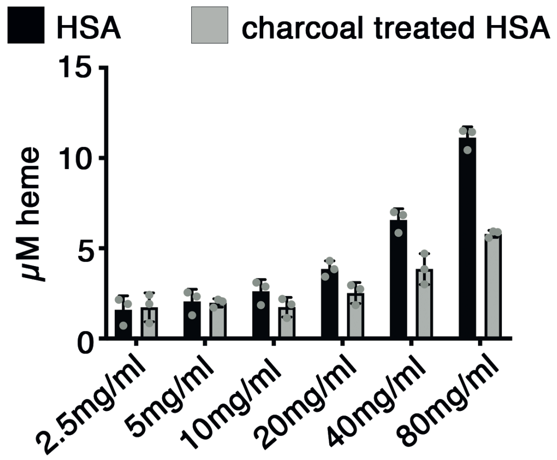

Determination of the heme concentration in HSA proteins. Data illustrate the heme concentration in HSA derived from human plasma. HSA and charcoal treated HSA (defatted HSA) was titrated and the protein bound heme concentration was measured via Heme Assay Kit. Data show mean ± SEM of three independent experiments.

Supplementary Figure 7

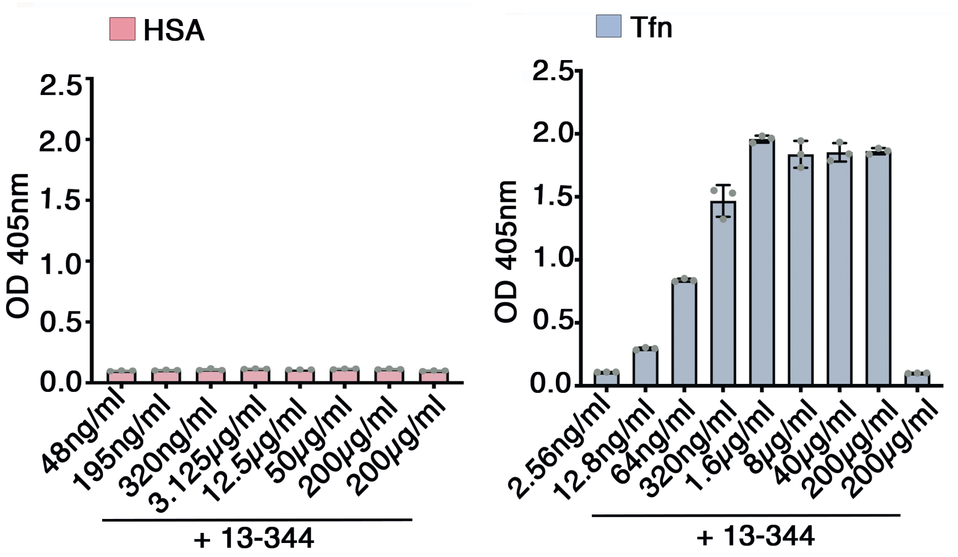

Analysis of transferrin in HSA. ELISA plate was coated with HSA or transferrin at titrated concentrations and incubated with an anti-transferrin antibody 13-344 (1 µg/ml). The binding of the antibody was measured at 405 nm with a photometer. Data are presented as means ± SD from one representative experiment, measured in triplicates.

Supplementary Figure 8

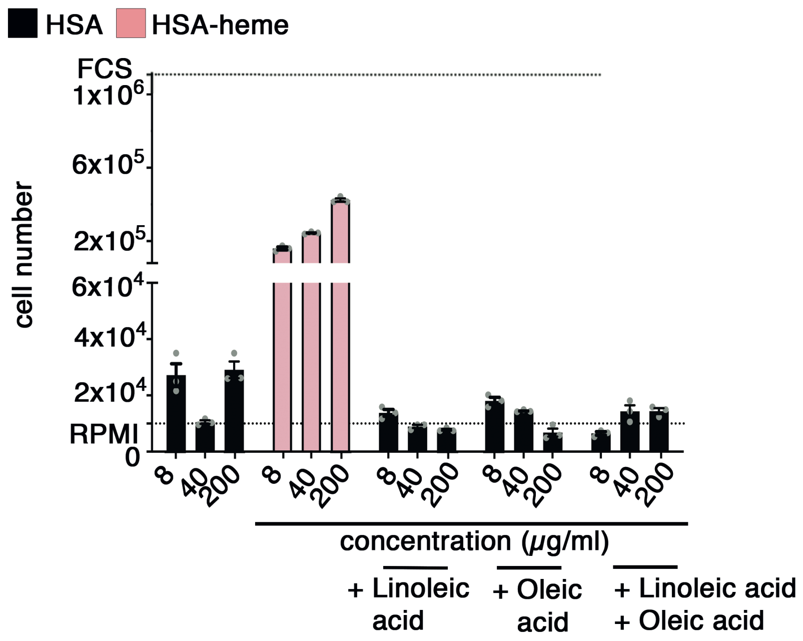

Effect of fatty acids on proliferation of Jurkat T cells. Cells were cultured with HSA or HSA-heme together with linoleic acid or oleic acid or in combination of both. Data represents one experiment of three.

Supplementary Figure 9

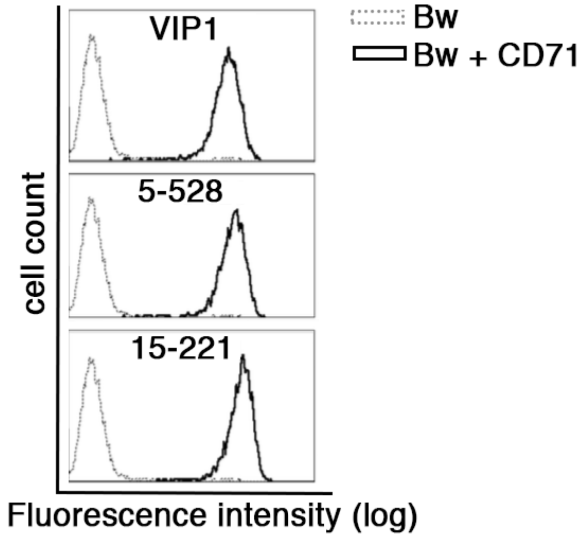

Analysis of the expression of CD71 receptor. Bw5147 cells and Bw cells transfected with human CD71 (Bw + CD71) were stained for CD71 expression. The CD71 mAbs VIP1, 5-528 and 15-221 were used for detection. Data shown are representative of two independent experiments.

Supplementary Figure 10

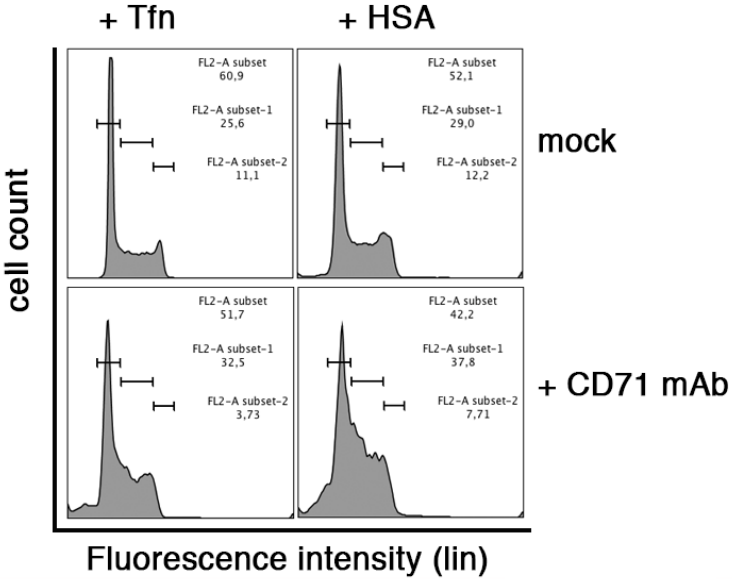

CD71 mAb blocks division of Jurkat T cells promoted by HSA. Cell cycle analysis of Jurkat T cells in presence of transferrin or HSA. Cell number was measured by flow cytometry after 4 days. Inhibition of cell proliferation with CD71 mAb VIP1 arrests cells in the S/G2-phase of the cell cycle in both cases. Data shown are representative of two independent experiments.

Supplementary Figure 11

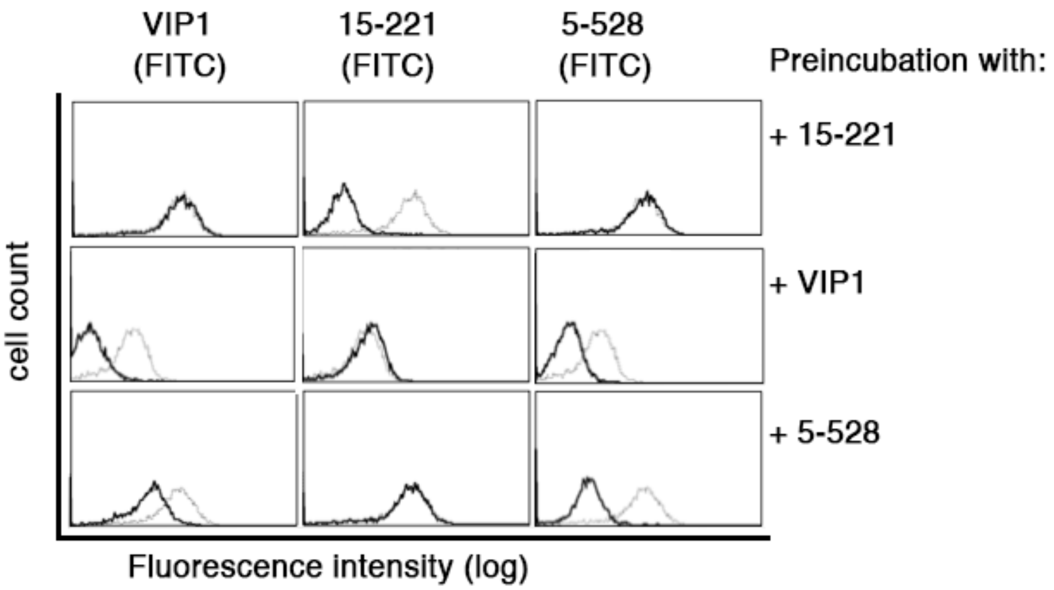

Analysis of overlapping epitope regions on CD71. Jurkat T cells were pretreated with CD71 mAbs VIP1, 15-221 or 5-528 (thick open histograms) for 30' at 4°C and additionally stained with FITC conjugated CD71 mAbs (dotted histograms). Data shown are representative of three independent experiments.

Supplementary Figure 12

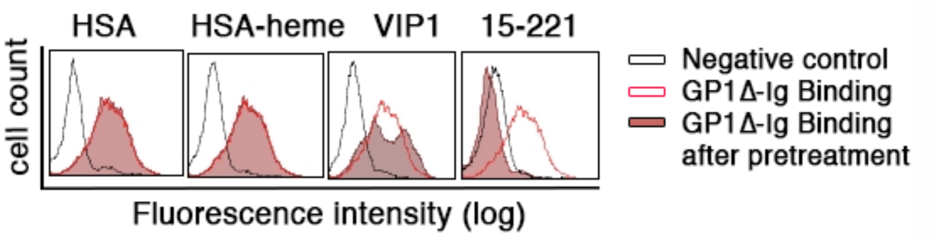

GP1Δ-Ig binding is inhibited by CD71 mAb 15-221 but not by HSA-heme. GP1Δ-Ig binding to CD71 was analyzed by flow cytometry. Jurkat cells were pre-incubated with HSA, HSA-heme or CD71 mAbs. Afterwards cells were incubated with GP1Δ-Ig protein. Data shown are representative of three independent experiments.

Supplementary Figure 13

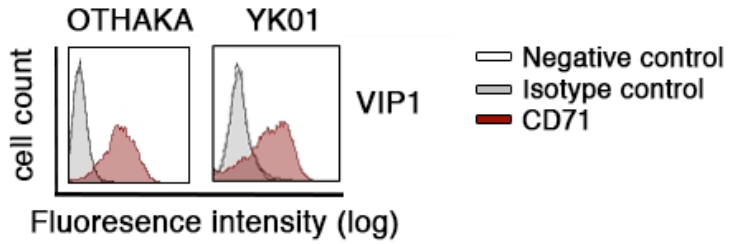

Expression of CD71 receptor on Epstein-Barr-Virus (EBV)-immortalized B cells. Identification of CD71 expression on EBV cell line OTHAKA with intact heme-oxygenase 1 HO-1 and YK01 cells with a defect gene. EBV cell lines were incubated with CD71 mAb VIP1 (5 µg/ml) or VIAP (isotype control). The expression profile was analyzed by flow cytometry.

Supplementary Figure 14

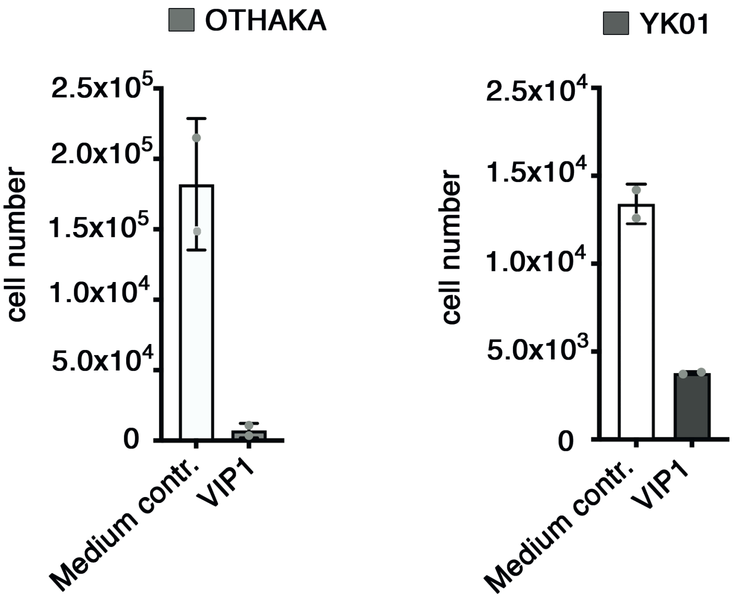

Growth of EBV cell lines enabled by transferrin can be blocked by CD71 mAb VIP1. Bar graphs show the proliferation of two EBV cell lines, which were incubated with CD71 mAb VIP1. Error bars indicate mean ± SEM from three independent experiments.
