# Supplementary material for: Transferrin receptor 1 is a cellular receptor for human heme-albumin
Source: Commun Biol. 2020 Oct 27;3:621. doi: 10.1038/s42003-020-01294-5 (PMC7591885; doi:10.1038/s42003-020-01294-5)
Supplement: Supplementary file 2 — Description of Additional Supplementary Files [file 42003_2020_1294_MOESM2_ESM.pdf]

Description of Additional Supplementary Files

File Name: Supplementary Data 1

Description: The source data behind graphs
