# Supplementary material for: Transferrin receptor 1 is a cellular receptor for human heme-albumin
Source: Commun Biol. 2020 Oct 27;3:621. doi: 10.1038/s42003-020-01294-5 (PMC7591885; doi:10.1038/s42003-020-01294-5)
Supplement: Supplementary file 4 — Reporting Summary [file 42003_2020_1294_MOESM4_ESM.pdf]

nature research

Corresponding author(s): Johannes Stöckl

Last updated by author(s): 2020 August 26

Reporting Summary

Nature Research wishes to improve the reproducibility of the work that we publish. This form provides structure for consistency and transparency in reporting. For further information on Nature Research policies, see Authors & Referees and the Editorial Policy Checklist.

Statistics

For all statistical analyses, confirm that the following items are present in the figure legend, table legend, main text, or Methods section.

n/a	Confirmed	
☐	☒	The exact sample size (*n*) for each experimental group/condition, given as a discrete number and unit of measurement
☐	☒	A statement on whether measurements were taken from distinct samples or whether the same sample was measured repeatedly
☐	☒	The statistical test(s) used AND whether they are one- or two-sided<br>*Only common tests should be described solely by name; describe more complex techniques in the Methods section.*
☒	☐	A description of all covariates tested
☒	☐	A description of any assumptions or corrections, such as tests of normality and adjustment for multiple comparisons
☐	☒	A full description of the statistical parameters including central tendency (e.g. means) or other basic estimates (e.g. regression coefficient) AND variation (e.g. standard deviation) or associated estimates of uncertainty (e.g. confidence intervals)
☒	☐	For null hypothesis testing, the test statistic (e.g. *F*, *t*, *r*) with confidence intervals, effect sizes, degrees of freedom and *P* value noted<br>*Give P values as exact values whenever suitable.*
☒	☐	For Bayesian analysis, information on the choice of priors and Markov chain Monte Carlo settings
☒	☐	For hierarchical and complex designs, identification of the appropriate level for tests and full reporting of outcomes
☒	☐	Estimates of effect sizes (e.g. Cohen's *d*, Pearson's *r*), indicating how they were calculated

Our web collection on statistics for biologists contains articles on many of the points above.

Software and code

Policy information about availability of computer code

Data collection	All datasets generated in the course of the current study are presented in the main text and the Supplementary information available online.
Data analysis	Graph Pad Prism 7 software was used in this study.

For manuscripts utilizing custom algorithms or software that are central to the research but not yet described in published literature, software must be made available to editors/reviewers. We strongly encourage code deposition in a community repository (e.g. GitHub). See the Nature Research guidelines for submitting code & software for further information.

Data

Policy information about availability of data

All manuscripts must include a data availability statement. This statement should provide the following information, where applicable:

- Accession codes, unique identifiers, or web links for publicly available datasets
- A list of figures that have associated raw data
- A description of any restrictions on data availability

All data generated or analysed during this study are included in this article and its supplemental information files. Further information about this study is available from the corresponding on request.

Field-specific reporting

Please select the one below that is the best fit for your research. If you are not sure, read the appropriate sections before making your selection.

☒ Life sciences ☐ Behavioural & social sciences ☐ Ecological, evolutionary & environmental sciences

For a reference copy of the document with all sections, see nature.com/documents/nr-reporting-summary-flat.pdf

Life sciences study design

All studies must disclose on these points even when the disclosure is negative.

Sample size	We aim at using n=5 as routine depending on if its primary cells or experiments with cell lines, but n=3 at least. Samples were collected from individual experiments from individual donors (mainly human but also murine cells). For cell lines samples were collected from individual experiments. No statistical method was used to predetermine sample size.
Data exclusions	No data was excluded.
Replication	Experiments were performed with biological and technical replicates, as described in the text. All data were reproducible.
Randomization	No such experiment were made.
Blinding	No such experiment were made.

Reporting for specific materials, systems and methods

We require information from authors about some types of materials, experimental systems and methods used in many studies. Here, indicate whether each material, system or method listed is relevant to your study. If you are not sure if a list item applies to your research, read the appropriate section before selecting a response.

Materials & experimental systems

n/a	Involved in the study	
☐	☒	Antibodies
☐	☒	Eukaryotic cell lines
☒	☐	Palaeontology
☒	☐	Animals and other organisms
☒	☐	Human research participants
☒	☐	Clinical data

Methods

n/a	Involved in the study	
☒	☐	ChIP-seq
☐	☒	Flow cytometry
☒	☐	MRI-based neuroimaging

Antibodies

Antibodies used	The following murine monoclonal antibodies (mAb) were raised in our laboratory: mAb VIAP (against calf intestine alkaline phosphatase), 5-528 (CD71), VIP1 (CD71), 15-221 (CD71), 13-344 (transferrin), mAb VIT6b (CD1a-PE), 5-272 (CD274-PE), MEM 18 (CD14-PE) and 7-239/44/0 (CD169-PE). APC-conjugated donkey anti-human IgG, goat anti-human IgG and anti-mouse conjugated alkaline phosphatase antibodies were purchased from Jackson-Immunoresearch Laboratories Inc. mAb OKT3 (CD3) was obtained from Janssen-Cilag (Vienna, AT). Human granulocyte–macrophage colony-stimulating factor (GM-CSF) and IL-4 were kindly provided by Novo Nordisk A/S (Bagsværd, DNK). MAb B7-2 (CD86-PE), mAb 10F3 (CD28), Oregon Green 488-conjugated goat anti-mouse IgG antibody and UltraPure EDTA was obtained from Invitrogen, UK. Anti-human beta-tubulin antibody (9F3-AF488) was acquired from Cell Signaling Technology Inc. (Frankfurt, DEU). Anti-human ferritin antibody (heavy chain, ferritin-AF647) was purchased from Santa Cruz (Dallas, US) and anti-human TFR antibody (TFR-AF647) from BD (Vienna, Austria).
Validation	The CD71 monoclonal antibodies used in this study were validated using cells from the murine BW-cell line expressing human CD71 molecules.<br>All other antibodies have been validated in previous publications.

Eukaryotic cell lines

Policy information about cell lines

Cell line source(s)	All human and murine cell lines used in this study are published and have a reference.
Authentication	The cells and cell lines were generated from the authors of this study or bought directly from commercial sources (ATCC) but

Authentication	not authenticated by us.
Mycoplasma contamination	The cell lines were routinely tested for mycoplasma and were negative.
Commonly misidentified lines (See ICLAC register)	No misidentified cell lines

Flow Cytometry

Plots

Confirm that:

- [ ] The axis labels state the marker and fluorochrome used (e.g. CD4-FITC).
- [ ] The axis scales are clearly visible. Include numbers along axes only for bottom left plot of group (a 'group' is an analysis of identical markers).
- [ ] All plots are contour plots with outliers or pseudocolor plots.
- [ ] A numerical value for number of cells or percentage (with statistics) is provided.

Methodology

Sample preparation	Flow cytometry was used in this study to analyze the marker profile of monocyte-derived dendritic cells, binding studies with CD71 ligands and heme-HSA, to monitor activation of NF-kB, AP-1, NFAT, for cell cycle analyses and detection of beta-tubulin by intracellular staining, as described in the Material and Methods section. These experiments were only performed with single cell populations or cells from cells lines and the results are presented as simple histograms or overlays.
Instrument	Data was collected on a FACSCalibur (Becton Dickinson) or LSRFortessa (Becton Dickinson).
Software	Analyses were performed with FloJo.
Cell population abundance	Purified cell populations (i.e. monocyte-derived DCs) or cells from cell lines were analyzed by flow cytometry in this study. At least 10.000 cells were analyzed per staining.
Gating strategy	Cells were gated on FSC and SSC area and PI staining to select living cells from debris. Unstained/background and isotype staining controls were used to determine positive gating.

- [ ] Tick this box to confirm that a figure exemplifying the gating strategy is provided in the Supplementary Information.
